# History of Medical Education and Institutions—Reviewing in General

**Published:** 1851-05

**Authors:** N. S. Davis

**Affiliations:** Chicago


### ARTICLE II.

*History of Medical Education and Institutions ; the New York Journal &c. ; Reviewing in General.* By N. S. DAVIS, M. D., Chicago, March, 1851.

"Far better would it be that new publications should pass unnoticed by the press, than that they should be spoken of only to be misrepresented. And we repeat that it makes no difference whether the misrepresentation be eulogistic or damnatory, for it is the right of the *reader* to be addressed *truly*, and of the *author* to be judged *fairly*. If the critic lose sight of his duty in these respects, he ceases to be a trustworthy guide to the public, or faithful to the literature of which he is a professed champion." See Dr. Stille's Report on Medical Literature, May, 185 0.

I make the above quotation as an appropriate introduction to a few remarks on the following notices of my recently published work on the "History of Medical Education and Institutions"; the first of which appeared in the January number of the N. Y. Journal of Medicine, under the head of "Critical Analysis." I give it to the reader entire as follows, viz.:

"The title of this work sounded so well in our ears, that we read it through from the beginning to the end. Having done so, we confess ourselves to have been grievously—most grievously disappointed. It is not what the title would indicate, or what we expected. Instead of being a fair and impartial history of what has been done in medicine in this country, it appears to us to be really nothing more than a tirade against quackery, and the various quackish practices which the author supposes to exist in our Medical Colleges. We shall, for a moment, glance at its contents. It consists of four chapters. The first is a "History of the Medical Profession and Medical Education in the British Colonies of America, from its first settlement to the achievement of their independence." This chapter occupies forty-eight pages, and is the only part of the work that has any pretentions to historical research. On looking over it, however, we find it nothing more than a crude compilation, altogether drawn from the writings of two or three authors, who have taken the trouble to give us full and elaborate accounts of this period of our medical history. Dr. Davis has not added a single new fact, as far as we can see, and the jumble and errors of names and dates is such as to show that he is entirely ignorant of the original sources of information. Now and then, indeed, he ventures and expression of opinion concerning a paper or an essay, but it is almost always in the language of some previous writer.

"The second chapter embraces the history of medicine in this country from the year one thousand seven hundred and eighty-three, to one thousand eight hundred and six: Here, Dr. Davis having no guide, is completely in the dark. He is like a mariner at sea without a compass, or even a star to guide him. He sees nothing, or knows nothing that the profession did during all this period, and he accordingly winds up a chapter of nearly forty pages in the following words: "I have not been able to find a single volume on any branch of medical science or practice, published by an American physician, durthe first twenty years after the close of the Revolutionary war." (p. 82) This is a precious confession for the historian of our profession, and we give him full credit for his honesty! What will the reader think, however, of Dr. Davis' knowledge, or his competency for the task of an historian, when he is told that the period mentioned by him abounds in original publications, and indeed is one of the most interesting eras in the literary annals of our profession? Out of a score of publications which might be mentioned, we select a few as samples.

*Medical Inquiries and Observations.* By Benjamin Rush, M. D. Philadelphia. 1793 to 1798. 5 vols. 8 vo.

*An historical Account of the Climate and Diseases of the United States of America.* By William Currie, M. D. Philadelphia. 8 vo 1792.

*Collections for an Essay towards a Materia Medica of the United States.* By Benjamin Smith Barton, M. D.. Commenced in 1798.

*Observations on the Causes and Cure of the Remitting Bilious Fevers, &c.* By William Currie, M.D. Philadelphia. 1798. 8 vo. pp. 227.

*A Treatise on Yellow Fever.* By John B. Davidge, A. M., M. D. Baltimore. 1798. 8vo. pp. 65.

*Dissertation on Croup.* By John Archer. 1798. 8vo. pp. 46.

*Letters on Yellow Fever.* By Richard Bagley, M. D. New York. 1798. 8vo. pp. 100.

*Memoirs on the Yellow Fever in Philadelphia, &c.* By William Currie, M. D. Philadelphia. 1798. 8vo. pp. 145.

*On the Origin of Pestilential diseases.* By Charles Caldwell, M. D. Philadelphia. 1799. 8vo.

*A Memoir on Goitre, as it prevails in different parts of North America.* By Benjamin S. Barton, M. D. Prof. Philadelphia. 1800. 8vo. pp. 95.

*Memoir on the Analysis of the Black Vomit, ejected in the last stages of the Yellow Fever.* By Isaac Catherall, M. D. Philadelphia. 1800. 8vo. pp. 32.

*On Kine Pox, &c.* By Benjamin Waterhouse, M. D. Boston. 1800. 8vo. pp. 40.

*On Hydrophobia.* By James Mease, M. D. Philadelphia. 1801. 8vo. pp. 62.

*Medical and Physical Memoirs.* By Charles Caldwell, M. D. Philadelphia. 1801. 8vo. pp. 296.

*On Vaccination.* By John Redman Coxe, M. D. Philadelphia. 1802. 8vo. pp. 152.

*Elements of Botany, illustrated with thirty engravings.* By Benjamin S. Barton, M. D. Philadelphia. 1803. 8vo. pp. 552, etc., etc.

These were all strictly *original* in their character, not mere compilations or reprints of foreign books. They related to American subjects, and rose fresh from the medical mind of the country; and yet not one of these appears ever to have met the eye of Dr. Davis!

The third chapter embraces the history of medicine in this country, from 1806 to 1850. The whole of this long chapter (80 pages) relates to the various medical societies established in the United States, and gives nothing but a dry, and we should say tedious detail, in relation to their organization, all of which might advantageously have been despatched in half a dozen pages.

The last chapter is on "the present condition and wants of the profession, and the remedies of those wants." On this chapter we have little to say. It contains nothing but what we have for several years heard iterated and re-iterated from various quarters, until we have become quite tired of it.

In conclusion, we have to say, that if the profession in this country is really in the degraded condition in which it is represented in this work, it will require somebody with more intelligence and ability than Dr. Davis possesses to elevate its character. Dr. Davis may be, and we doubt not is, a wellmeaning man; but he was never born to be the Solon of our profession. Let him study a little more; let him get a little more knowledge in relation to the past and present history of our profession, and then let him come forward and correct its defects. At present, we must pronounce his book a perfect *caricature*, and a *libel* upon a profession, which, notwithstanding all its short comings and deficiencies (and of these we are fully sensible), is yet eminently respectable.

We have been forced to these remarks from the necessities of the case, and the remembrance of the use which is made abroad of the imperfect histories of the American profession and its literature, written by incompetent persons. We have seen with what eagerness our brethren across the Atlantic have seized hold of the shameful retrospect of our literature by Dr. Holmes, Chairman of the Committee on Medical Literature for the American Medical Association for 1848, and this, too, notwithstanding the protest we urged in the March No. for 1849 of this Journal, showing the instances repeated and numerous, the inaccuraces and inconsistencies of that "Retrospect"—and we have seen, we continue, the meanness of our calumniators, and are resolved that our approbation shall not be placed upon anything but the truth, *and the whole truth.*"

On the above the New York Medical Gazette comments as follows, viz:

"A Critical Analysis of Dr. Smith's great work on Neuroma, and Dr. Davis' *little* work upon Medical Education, &c., are both able and *judicious*."

On the same, the Western Journal of Medicine and Surgery, published at Louisville Ky., has the following:

"Our confrere of the New York Journal of Medicine gives Dr. Davis a severe castigation for the work dignified with the *title* of *History of Medicine*. We think the profession generally will coincide with the sentiments of the Reviewer. Dr. Davis' History of Medicine is neither a work of information nor accuracy. Instead of being a history of Medical Institutions, it is a gross caricature of the subject. It proclaims deficiency where none exists, and assails the profession in a style that is neither truthful nor useful. The man who rises from a perusal of Dr. Davis' History, under the impression that he has read the details of American Medical History, will make as great a mistake as he would in supposing that he had read the history of America, in reading Paulding's History of John Bull and Brother Jonathan."

Finally, the March number of the "Western Medico Chirurgical Journal," published at Keokuk, in the Southeastern part of the State of Iowa, introduces the above comments of the Western Journal to its readers, with the following dignified remarks:

"*Dr. Davis' History of Medicine.* This gentleman seems to have been singularly unfortunate in his efforts to acquire notoriety and fame. Some years ago, as Editor of a small medical pamphlet, published in New York, he sought distinction by advocating some wild eccentric notions of medical education, and by *keeping up a constant abuse and defamation* of the regular medical profession of the United States. Finding his efforts unavailing, *and receiving nothing but contempt* for his labor, he sought and obtained a place in a Medical School at Chicago, Ill., where he has been manufacturing "Doctors" at wholesale during the past two years, and contradicting all his former pretensions, by extending an indiscriminate privilege to every one who chose to enter himself for the degree."

This last quototion is made solely for the purpose of showing our readers, and especially my old friends at the east, with whom I have acted and toiled, through many a pleasant session of the New York State Medical Society, and the American Medical Association, a rare specimen of *fraternal* regard for *ethical propriety*. Personal abuse and vulgar detraction generally proceed from an empty head, or a bad heart; two things wholly unworthy the notice of an industrious and upright man. Hence the remarks which follow will be directed exclusively to the Notice of the New York Journal of Medicine, and that founded on it in the Western Journal, as quoted above.

There are three qualities essential to all criticisms worthy of the name. First, the critic must represent fairly and truthfully, the *objects*, *sentiments*, and *style* of the work criticised. Second, he must give such an analysis as will convey to his readers a just idea of what the work actually contains. Third, his own style should be free from a spirit of petulence or personal detraction. These being rules about the correctness of which there can be no dispute, let us apply them to the above criticism of the New York Journal of Medicine. And first, does the writer fairly and *truthfully* represent the *objects* of the book under review? Does he carefully ascertain from its title and subsequent pages, its general scope and tendency, and then inform his readers correctly in regard to the apparent *intentions* of the author? He says: "Instead of being a fair and impartial history of what has been done in medicine in this country." Again: "The second chapter embraces the *History of Medicine*," &c. And again, "The third chapter embraces the *History of Medicine* in this Country," &c. Thus conveying to the reader, most fully, the idea that the book under review, claims to be a History of Medicine in this country; and actually inducing the Editor of the Western Journal to speak of it as "*the* work *dignified* with the *title* of *History of Medicine*." It has generally been supposed, that a History of Medicine must consist essentially of a narrative of the chief facts, circumstances, and opinions, connected with the progress of Medical Science and practice.

Hence, we find the Medical Historian, from the days of LeClerc to the present time, tracing the progress of medical opinions or theories, medical and surgical discoveries and improvements, and the names of eminent medical men connected therewith; with little or no reference to the particular laws, societies, or schools of any given country. But neither the title page, preface, nor any single paragraph in the whole volume of the work under review, makes any pretentions to the character of a "History of *Medicine* in this country." On the contrary, the author in more than one place, expressly disavows any intention of making his work embrace the subjects belonging to such a history. Thus on page 35, he says: "Although a consideration of *medical practice does not* come strictly within the scope of our present work, yet an *occasional* glance at this, and the character of diseases prevalent at different periods of time, will be both interesting and profitable." And again on page 155, "In the two preceeding chapters we have given a *brief* account of the medical works published, and of the improvements made during each period; but even a *simple enumeration of these*, during the last forty years, would extend the limits of this work too far, and contribute but *little to its main design*." What then was the object of the author? Just what his title page, and the heading of each chapter of the work would indicate, viz: to present a history of Medical *Education* and *Institutions*; embracing an account of the medical laws, societies, and schools in this country, and their influence on the interests and character of the profession; an object which stands out in full view on every page of the book. And yet, with the title page directly before him, the reviewer proceeds scarcely more than three lines before he entirely misrepresents that title, and then, on such misrepresentation, founds his most sweeping condemnation of the whole work. In this, he stands fully convicted of neglecting the very first duty of the critic, by which his whole performance is vitiated and shown to be unjust.

Having thus perverted the title and objects of the work, it is not strange that he should declare its *sentiments* "*to be really nothing more than a tirade against quackery, and the various quackish practices which the author supposes to exist in our Medical Colleges*." An assertion, the truthfulness of which, may be judged by the fact, that scarcely one of the various systems of quackery prevalent at the present time, is so much as named in the book, and on none of its pages is so vile an epithet as "*quackish*," applied to, even the most objectionable *practices* of the medical schools. But this part of my task will be presented more fully under the application of the second rule stated above, viz: that the reviewer should give such an analysis of the work, on the merit of which he assumes to sit in judgment, as will convey to his readers a just idea of what it actually contains. Does the New York Journal critic do this?" Let a reference to his comments on the second and third chapters of the book answer. In reference to the former, he asserts that the author, "*sees nothing*, or *knows nothing* that the profession did during all this period, and he accordingly winds up a chapter of near *forty* pages in the following words."

This is the sum and substance of his *critical analysis*, of chapter second; and what is the impression it conveys to the reader? Simply, that I have written near forty pages to arrive at the grand conclusion, that during the first twenty years after the revolutionary war, nothing was done in medicine, not so much as the publication of an original memoir by an American Physician. Sure enough! "what will the reader think," not of my competency or incompetency, but of the fairness and honesty of the reviewer when he turns to the chapter referred to, and finds the greater part of it in strict accordance with the title and objects of the whole work, devoted to an account of the establishment of Medical schools and hospitals, the formation of Medical Societies, the establishment of the first Medical Journals in New York and Philadelphia, and the laws relating to medical education, during the first quarter of a century after the Revolution; while the remaining portion alludes briefly to the medical *writers* and *writings* of that day. Yes, notwithstanding the total darkness in which it was written, the chapter does allude to *writings* and *publications*, and that too in no disparaging terms. Thus after alluding to the names of a Warren, Waterhouse, Bayley, Bard, Miller, Mitchell, Post, Romaine, Middleton, Jones, Rush, Barton, Shippen, Dewees, John Mitchell, Chalmers, Moultrie, and others, as constituting "a bright galaxy on the pages of American Medical History," it asserts that, "Many of these were not only learned, but they were characterized by a bold, free spirit of inquiry, which soon broke through the theoretical dogmas of that day, and did more by *their writings* to overthrow the absurdities of the Boerhaavian school than any equal number of cotemporaries on the other side of the Atlantic." (see pp. 69.) And strange as it may seem, the "Medical Inquiries and Observations" of Dr. Rush, which the reviewer mentions as never *having met my eye*, are not only referred to by name, but whole paragraphs are quoted from them with due credit. But if there were writings and publications during the period embraced in the chapter, what is the meaning of those four lines of "Precious Confession"? The following quotation containing what precedes and follows those lines, will furnish an answer. "The medical writings of this period seem to have been confined almost wholly to the pages of the three medical journals which had been established in New York and Philadelphia, and to here and there a pamphlet, or a paper read before some organized society. Indeed, I have not been able to find a single *volume* on any *branch* of medical science or practice, published by an American physician during the first twenty years after the close of the revolutionary war. And yet, as we have already seen, it was a period in our history which was graced by some of *the noblest and most active minds ever devoted to the cultivation of medical science.* But the art of *mere book making*, which has been brought to such perfection in this our day, was little known to our professional ancestors." Such is the connection in which the "precious confession" stands in the book.

And what is its fair and legitimate meaning? That I had not been able to find a single *memoir*, dissertation, pamphlet, or even *volume*, on special or detached medical topics, published by American physicians, as represented by the reviewer? Certainly not; for the existence of these is not only admitted in the immediately preceeding paragraph, but numbers of them had been alluded to, and some quoted from throughout the chapter. The true meaning which seems to me obvious to the most careless reader, is, that I had been unable to find a volume, published within the period specified, on any

"*branch of medical science*," i. e., on Physiology, Midwifery, Materia Medica, Practical Medicine, &c., and I verily believe still, that it will puzzle the reviewer himself to find any such volume.

But if the author was thus sitting in total darkness, without "even a star to guide him," the curious reader must certainly be interested in knowing what the "*forty pages*," written under such peculiar circumstances, could relate to. And it was at least cruel, if not unjust, for the critic to entirely withhold such information. His *analysis*, however, of the third chapter is still more laconic. Here it is again: "The whole of this long chapter (80 pages) relates to the various medical societies established in the United States, and gives nothing but a dry, we should say tedious detail, in relation to their organization, all of which might advantageously have been despatched in half a dozen pages." Here, surely, is exhibited a power of condensation without a parallel. A chapter of 80 pages is *critically analysed* in the compass of *four* manuscript lines. But, I must ask again, in all candor, "what will the reader think" of the reviewer's regard for *truthfulness*, when he learns that, instead of having devoted "*the whole*" of this long chapter to "*nothing*" but an account of medical societies; no less than *thirty-three* of the eighty pages contained in it, are occupied with an account of medical colleges, medical laws not relating to societies, and to valuable contributions to medical science and practice; leaving 46, only, devoted to an account of social organizations, 34 of which relate exclusively to the origin, constitution, progress and doings of the American Medical Association? And yet the reviewer makes the unqualified and emphatic assertion to his readers, that "*the whole*" chapter "*gives nothing*" but a dry detail concerning medical societies.

Is it possible, that such wholesale misrepresentations can serve any useful purpose, either at home or abroad? Will they elevate the character of the profession, or encourage and foster its literature? And will they do so any the more effectually because him who makes them pompously assures us that *his* "approbation shall not be placed upon anything but the *truth*, and the *whole truth?*" Concerning the last chapter, and the general *style* of the work, the reviewer declines to give his readers any information; but as if impatient of the time he had already wasted on so worthless a book, he hastens to announce the final result of his examination as follows, viz:

"In conclusion, we have to say, that if the profession in this country is really in the *degraded* condition in which it is represented in this work, it will require somebody with more intelligence and ability than Dr. Davis possesses to elevate its character.

"At present, we must pronounce his book a perfect *caricature*, and a *libel* upon a profession, which, notwithstanding all its short comings and deficiencies (and of these we are fully sensible), is yet *eminently respectable.*" Following in the same strain, the Louisville editor says:—"It proclaims deficiency where none exists, and assails the profession in a style that is neither *truthful* nor *useful.*" Here, then, the book is distinctly charged, not only with *assailing* and representing the profession as in a "*degraded*" condition, but with being a "*cariture*" and a "*libel*" upon it. These are grave charges; and when proclaimed in a manner to be read on both sides of the Atlantic, they should be accompanied by some substantial proof of their *truth;* and that, too, even though they affected the character of no one but him who was once the "*young man in the township of Binghampton.*" But where is the proof given to the readers of the above journals? Do they quote a paragraph from one of those *libelous* pages, or even a single line of that "*tirade*" against the profession, and thereby enable the reader to judge for himself? Not at all; but they leave the naked editorial, *ipse dixit*, standing out with as much gravity as though it was not only, like the laws of the Medes and Persians, *unalterable*, but also *indisputable*. Having already shown this authority, however, to be at least *fallible*, I shall hazzard an explicit denial of these charges in all their length and breadth, and hold their authors as guilty of most inexcusable injustice until they adduce their proof. The *New York Journal* critic charges the book with representing the profession as in a "*degraded condition*." I ask him, where? Is it in the beginning of the work, where it is asserted thus: "The medical profession in the United States, and, indeed, throughout the civilized world, constitutes an important part of Society; for while, on the one hand, its ranks can boast not only of names of the highest eminence in every department of science and literature, but can claim to be equal with the foremost in every enterprise for extending human knowledge, and ameliorating human suffering, its free access to the homes and firesides of all classes, gives it a moral and social influence of the most potent character. *And in no part of the world is this influence more extensively or happily felt than in this country*, where the absence of all hereditary distinctions and privileged orders, leaves learning and virtue free to assume her own native eminence."

Or is it at the close of the historical part of the work, where the author asserts; that, "notwithstanding the abolishment of all laws regulating the practice of medicine in so large a number of States, and the consequent absence of all legal protection, the profession was, probably, never making more rapid advancement in its education, its science and literature, and its *social position*, than at the *present* time.

And that the profession of no country have made, during the same length of time, more *numerous* or *valuable* additions to our resources for combating disease and alleviating pain than that of our own." Yet, the critic of the *New York Journal* gravely asserts that the profession in this country "is eminently *respectable*;" just as though the work subjected to his examination denied such *respectability*. An *insinuation* as *unworthy* a candid mind, as many of the reviewer's previous assertions are false. No man entertains a more profound respect for the medical profession, or a warmer attachment to all its legitimate interests, than the author of the book under review. But I trust that no degree of *respect* will ever make him so blind or unfaithful to the cause of truth, as to deny the existence of those errors and defects which have so long marred its education, diminished its usefulness, and retarded all its interests. And much less will he, like the New York critic and his associates, assume the ill-natured position of "*the dog in the manger*," by declaring that he is "*fully sensible*" of all its "*short comings* and *deficiencies*," and yet, instead of pointing out what they are, and how they may be remedied, spend his time in petulent *snarls* at whoever attempts the task.

It is true that my work on the History of Medical Education, &c., represents the profession as containing many members whose education, both preliminary and medical, is extremely deficient; that its practical standard of preliminary education is too low; that the college terms are altogether too short in proportion to the number and extent of subjects embraced in them; that a large number of the schools are so located as to preclude the possibility of giving their classes the advantages of hospital or bed-side instruction; that the present system of examinations for the degree, is wholly inadequate to test the qualifications of the candidate; that, in many of the States, a large proportion of those who commence practice do so without graduating anywhere; and, finally, that the social organization of the profession is incomplete. These are the *deficiencies* which "*it proclaims*." And will our friend of the *Western Journal* be kind enough to inform us and his readers, which of them *does not exist?* Will he, at the same time, point to the page on which the profession is

*assailed* in a "style that is neither *truthful* nor *useful*;" or else take back the unfounded assertion?

It was stated in the beginning, as the third essential rule of criticism, that the critic's own style should be free from a spirit of petulence or personal detraction. But the *New York Journal* reviewer will bear the application of this scarcely better than either of the two which preceded it. There is, in much of his phraseology, an air of petulence, bearing, indeed, a near approach to overbearing *insolence*, which is wholly unbecoming a scientific and professional mind. Much as I am accused of *calumniating* the profession, in no part of my writings can words so exceptionable as "*shameful*"—"*tirade*"—"*libel*," and "*meanness*," be found applied even to the humblest practitioner in America. For the reviewer's kind advice "*to study a little more—to get a little more knowledge*" &c., I am certainly thankful. It is only seventeen short years since I began acting in accordance with this advice, and though, during that time, I have neither shunned the midnight lamp, nor the noon-day sun; neither avoided in my search the flower-decked hill side, the noisome laboratory, nor the warm gushing blood of animals sacrificed to science; yet, *encouraged* by the kind notices of my labor which have just passed in review, I shall certainly double my dilligence and renew the pursuit. But so long as my love for the profession remains undiminished, there is one thing I cannot, *will not* do; and that is, to cease urging such measures as I think calculated to elevate its character and extend its usefulness; even though, in so doing, I might chance to gratify the national spleen of some testy critic on the other side of the Atlantic.

Having now shown that my friend of the *New York Journal*, and, of course, his Western coajutors, have been guilty of transgressing every fair and legitimate rule of criticism, in their notices of my book, I have only to enquire in conclusion, how *such* notices are to improve our medical literature, or, in any way, advance the interests of the profession?

All acknowledge that our system of medical education has defects, both numerous and important. None are so bold as to deny this. Yet, for several years past, there have not been wanting men of influence, holding the responsible and advantageous position of editors, who, instead of candidly pointing out these defects and faithfully suggesting remedies therefor, have never failed to promptly and most authoritatively condemn every attempt of others to do so. This does not result so much from personal enmity or private interest, as from a thoughtless disregard of duty.

No department of journalism is more vitally important to the welfare of the profession than that relating to reviews and notices of books. It is here that the editor or critic sits in judgement on the labors of his brethren, and presumes to decide for his readers what is worthy of their attention and what not. Hence he is bound to bring to his task, at all times, that rigid regard for truth and fairness, that unbending impartiality, and that stern sense of justice, which should ever characterize the position of judge. And no circumstances of personal friendship or petulent disappointment, can excuse him for indiscriminate praise, on the one hand, or wholesale and even overbearing censure on the other. What encouragement has any man to use his time and the little talent which his creator has given him, in efforts to improve the character and extend the usefulness of his profession, if, before his work can reach a dozen readers, the review department of our most influential journals can be used, not only to pervert the plain objects of his labor, but to sweepingly damn his whole performance as "a *caricature and a libel*," without so much as quoting a single page to enable the reader to judge for himself? Will the pitiful acknowledgement that the author is doubtless "*a well-meaning man*," compensate for injustice like this? Or will the fear of furnishing materials to gratify the prejudices of some *foreign* review, excuse the act? Away with such puerile nonsense. If we have confessedly "*short*

*comings* and *deficiencies*" in the profession, let us frankly acknowledge them, and then set ourselves at work, like honest and independent men, to devise ways and means for their removal. I have taken the trouble to pen this article, not in self-defence or from personal considerations, but simply for the promotion of truth and justice. For, though I make no pretensions to a stoical indifference to the opinions of my professional brethren, yet I am not conscious that either the desire of praise or the fear of censure, will ever deter me from speaking and writing whatever I deem calculated to advance the interests of my profession or the welfare of mankind.

CHICAGO, March 27, 1850.

